# A novel 3D culture model for human primary mammary adipocytes to study their metabolic crosstalk with breast cancer in lean and obese conditions

**DOI:** 10.1038/s41598-023-31673-x

**Published:** 2023-03-22

**Authors:** Marie Rebeaud, Caroline Bouche, Stéphanie Dauvillier, Camille Attané, Carlo Arellano, Charlotte Vaysse, Frédérique Fallone, Catherine Muller

**Affiliations:** 1 Institut de Pharmacologie et de Biologie Structurale, CNRS/Université de Toulouse UMR 5089, 205 route de Narbonne, BP 64182, 31077 Toulouse, France; 2 Département de Chirurgie Gynécologique oncologique, CHU-Toulouse, Institut Universitaire du Cancer de Toulouse-Oncopole, 1 avenue Irène Joliot-Curie, 31059 Toulouse Cedex 9, France

**Keywords:** Biological techniques, Cancer

## Abstract

Obesity is a negative prognosis factor for breast cancer. Yet, the biological mechanisms underlying this effect are still largely unknown. An emerging hypothesis is that the transfer of free fatty acids (FFA) between adipocytes and tumor cells might be altered under obese conditions, contributing to tumor progression. Currently there is a paucity of models to study human mammary adipocytes (M-Ads)-cancer crosstalk. As for other types of isolated white adipocytes, herein, we showed that human M-Ads die within 2–3 days by necrosis when grown in 2D. As an alternative, M-Ads were grown in a fibrin matrix, a 3D model that preserve their distribution, integrity and metabolic function for up to 5 days at physiological glucose concentrations (5 mM). Higher glucose concentrations frequently used in in vitro models promote lipogenesis during M-Ads culture, impairing their lipolytic function. Using transwell inserts, the matrix embedded adipocytes were cocultured with breast cancer cells. FFA transfer between M-Ads and cancer cells was observed, and this event was amplified by obesity. Together these data show that our 3D model is a new tool for studying the effect of M-Ads on tumor cells and beyond with all the components of the tumor microenvironment including the immune cells.

## Introduction

Breast cancer (BC) is the most common cancer and the second leading cause of cancer-associated death among women worldwide^1^. Mammary adipose tissue (MAT) represents a major component of the BC microenvironment, and numerous studies now indicate that MAT adjacent to tumors supports BC development and progression^2^. A large number of studies supports the existence of a bidirectional crosstalk between BC cells and mammary adipocytes (M-Ads), the major cellular component of MAT, at the tumor invasive front (for review^3–5^). Coculture models using adipocytes (obtained from in vitro differentiation of murine pre-adipocyte cell lines or ex vivo differentiated adipose progenitors) have been used to provide mechanistic insights of this deleterious dialog (for review^3–5^). Adipocytes affect cancer aggressiveness through soluble factors such as pro-inflammatory cytokines^6^, extra-cellular matrix (ECM) proteins and proteins involved in ECM remodeling^7,8^ or through lipid transfer affecting tumor metabolism^9^ (for review^3–5^). One of the most specific and emerging mechanism regarding the role of adipocytes involves the ability of cancer cells to hijack the nurturing role of adipocytes, the largest reservoirs of lipids in the tumor microenvironment (TME), to their advantage^4^.

Epidemiological arguments suggest that this dialog might be amplified in obesity, where the secretory and metabolic profiles of adipocytes are affected^10,11^. Regardless of menopausal status, obesity worsens the prognosis of BC^12,13^. BC mortality increases by 18% for every 5 kg/m^2^ increase in body mass index (BMI), and the risk of relapse at 5 years in obese patients is of 40% compared to 5–10% in unselected populations^12^. Understanding the biology and mechanisms of this effect will provide a timely opportunity for improving the treatment and outcomes of obese patients with BC.

To date, most studies have focused on studying changes in adipokines secretion and/or chronic inflammation in MAT of obese subjects with BC^14^. Increased lipid transfer between M-Ads and cancer cells might be a key event promoting BC aggressiveness in obesity, an hypothesis that remains largely unexplored^4^. In obesity, mature adipocytes are larger in size due to an increased need to store lipids as triglyceride (TG) in their lipid droplet (LD)^10^. As for other fat depots, we and others have shown that M-Ads also increase in size in overweight and obese patients, and that there is a positive correlation between M-Ads size and BMI^15–17^. Lipolysis, a process corresponding to the hydrolysis of TG into free fatty acids (FFA), that are then released in the extra-cellular medium, is also affected by obesity^18^. An increase in basal and a decrease in catecholamine-stimulated lipolysis have been described in various fat depots^18^, although these modifications have never been studied in M-Ads. Recent lipidomic profiling of human subcutaneous and visceral adipose tissue (AT) of lean and obese individuals has revealed qualitative and quantitative differences of the lipid content with obesity, and also between different adipose depots^19^. Accordingly, using M-Ads isolated from lean and obese patients is fundamental to study the metabolic crosstalk between BC cells and mature adipocytes in the context of obesity under the most suitable conditions.

To our knowledge, no validated experimental model exists to answer this question. Although mature adipocytes can be easily isolated by flotation after collagenase digestion, they are not able to survive for more than 24 to 48 h in culture medium^20,21^. Due to their high lipid content, adipocytes float on the top of the culture medium resulting in dedifferentiation (leading to the appearance of elongated fibroblast-like cells), delipidation and ultimately, cell death^20–22^. A recently published protocol described a 2D culture model of primary M-Ads over several days^23^ suggesting that these cells behave differently than other adipocytes. However, we found in our current study that in these conditions, M-Ads, like sub-cutaneous adipocytes (SC-Ads) undergo rapid loss in cell viability as shown in the results section. A more appropriated approach could be to culture isolated mature adipocytes in semi-solid matrices. Several types of 3D models have been reported using mostly SC-Ads embedded in collagen, Matrigel or hyaluronan-based hydrogel^20^. These approaches support adipocyte viability for several days but some pitfalls can be pointed out. In collagen matrices, that are semi-liquid at 37 °C, a significant proportion of adipocytes undergo an almost general delipidation after 1 week^24^. Culture in Matrigel, that is extracted from the basement membrane from an Engelbreth–Holm–Swarm mouse sarcoma^25^, maintains isolated adipocytes intact for up to 6 days^26^, but soluble factors contained in the ECM of murine tumors could affect the behavior of cocultured BC cells and introduce variability in the experimental results^25^. Finally, a model of adipocyte aggregates cultured under a membrane (Membrane Adipocytes Aggregates Culture or MAAC) has been recently proposed to maintain adipocyte morphology and function for up to 7 days^27^. To our knowledge, few of these culture systems have been used to maintain adipocytes isolated from obese patients. Studies have proposed to “recreate” the obesity setting by differentiating adipose progenitors in 3D culture systems and to expose them to exogenous FFA to mimic caloric overload^28,29^. However, these models might not appropriately reflect the quantitative and qualitative nature of the accumulated lipids present in M-Ads during obesity, since their nature vary depending on the adipose depot^30^**.**

To circumvent these problems, we set-up a 3D culture system of M-Ads embedded in a fibrin matrix that has been previously used to culture SC-Ads over a short time^31^. Fibrin has the advantage of polymerizing quickly, which allows a homogeneous distribution of adipocytes in the gel, preventing them from rising to the upper part of the gel during the polymerization phase, an event that favors delipidation, as observed with collagen matrices^24^. By optimizing the glucose concentration in the culture medium, we described here a novel 3D culture system of M-Ads obtained from lean and obese patients, enabling maintenance of their cell integrity, size and stimulated-lipolytic function for up to 5 days. In coculture with BC cells, we validated the use of this model to study the transfer of FFA between M-Ads and cancer cells, and our preliminary results showed that this process is increased by obesity. This model represents a new tool to investigate the role of increased lipid transfer between surrounding M-Ads and cancer cells in BC aggressiveness in obese patients.

## Materials and methods

### Tissue collection

Mammary adipose tissue (MAT) samples were collected from patients undergoing mastectomy for BC at the Toulouse-Oncopole University Cancer Institute (IUCT-O) between January 2020 and September 2022 (Toulouse, France). The study was conducted in accordance with the guidelines and with the full approval of the national CODECOH committee (authorization AC-2016-2658, DC-2016-2656). Written informed consent was received from participants before inclusion in the study, which was conducted in accordance with the Declaration of Helsinki principles as revised in 2000. MAT samples were collected in the quadrant opposite to the tumor, at a distance of at least 3 cm from the tumor. The patients with a history of homolateral breast surgery, chemotherapy, breast and/or axillary radiotherapy, hormone therapy were excluded from the study. Samples were either obtained from normal weight (NW) (BMI between 18.5 and 25 kg/m^2^) or obese patients (BMI greater than 30 kg/m^2^). For 2D coculture, subcutaneous adipose tissue (SC-AT) were obtained from NW women undergoing hip surgery at the Orthopedic Surgery and Traumatology Department of the Hospital Pierre Paul Riquet (Toulouse, France). All patients gave informed consent, and the samples were obtained according to national CODECOH committee (authorization DC-2017-2914). Samples were immediately put in 50 mL tubes containing 10 mL of Dulbecco’s Modified Eagle’s Medium (DMEM) (Thermo Fisher Scientific,) and carried out to the research lab within 1 h.

### Adipocyte isolation

Adipocytes were isolated as previously described^32^. Briefly, after cutting them into small pieces, MATs (5 to 15 g) were digested with type I collagenase from Sigma-Aldrich at 250 U/mL in PBS containing 2% bovine serum albumin (BSA) for 30 min at 37 °C, under shaking. After digestion, the cell suspension was filtered through a 200 µm strainer to remove cell debris and undigested fragments. Floating adipocytes were collected and washed several times with KRBHA (Krebs–Ringer Bicarbonate buffer from Sigma-Aldrich supplemented with 10 mM HEPES and 0.5% BSA pH 7.4) to obtain pure isolated adipocytes.

### BC cell line and culture

The human BC cell line MDA-MB-231 (provided by C. Dumontet, CRCL, Lyon, France) was cultured in RPMI medium without glucose (Thermo Fischer Scientific) supplemented with 5 mM glucose (Sigma Aldrich), 5% fetal calf serum (FCS) and 1% Penicillin–Streptomycin (P/S). Cells were grown at 37 °C in a humidified atmosphere with 5% CO_2_ and used within 2 months after resuscitation of frozen aliquots. The cells were tested every month by polymerase chain reaction for mycoplasma contamination.

### 2D culture of adipocytes

2D culture was performed according to the protocol of Picon-Ruiz et al.^22^. Briefly, after isolation, 1 mL of adipocytes were resuspended in 5 mL of DMEM supplemented with 10% FCS and 1% P/S, and were distributed in a 6-well plate (2 mL per well) (corresponding to approximately 6 × 10^5^ cells per well). Adipocytes were maintained in culture for 7 days at 37 °C in a humidified atmosphere with 5% CO_2_. Every 2 days, 500µL of DMEM was added to each well. To monitor their viability, pictures of the adipocytes were taken at D0, D3 and D7 with a light microscope (Olympus CKX53) and refringent cells were numbered using a Malassez counting chamber. Viability was also assessed after staining with BODIPY® 493/503 and measure of the lactatate deshydrogenase (LDH) concentration in the supernatants (see below).

### 3D culture of adipocytes

To prepare the 3D fibrin matrix, 100 µL of isolated adipocytes (corresponding to approximately 2 × 10^5^ cells) were first put in 24-well plates. Then, for “undiluted” or “diluted” gels, respectively 100 µL or 70 µL of fibrinogen at 18 mg/mL (Sigma Aldrich) in NaCl solution were added to the wells (respectively 6 and 4.2 mg/mL final concentration) and were gently homogenized. For "diluted" gels, 30 µL of culture medium was added to obtain the same culture volume. One hundred µL of thrombin (Sigma Aldrich) at 25 UI/mL in PBS-CaCl_2_ were added and homogenized quickly. Finally, gels were placed at 37 °C for a few min to polymerize. One mL of culture medium (DMEM containing 25 mM glucose with 10% FCS and 1% P/S or RPMI 1640 containing either 5 or 11 mM glucose with 5% FCS and 1% P/S) was then added to each well. All the media were purchased from Thermofisher. Gels were kept for 5 days at 37 °C in a humidified atmosphere with 5% CO_2_.

### MDA-MB-231 coculture with adipocytes embedded in 3D matrix

Tumor cells and adipocytes were cocultured using a transwell culture system (0.4 µM pore size; Dutscher). MDA-MB-231 BC cells were seeded on glass coverslips at 1 × 10^4^ cells per well in the lower chamber of 24-well plates, and isolated adipocytes embedded in 3D fibrin matrix were seeded in the upper chamber of the 24-well transwell inserts. Five hundred µL of RPMI supplemented with 5 mM glucose, 5% FCS and 1% P/S were added to both lower and upper chambers. After 3 days of coculture, cancer cells were stained with DAPI, rhodamine phalloidin and BODIPY® 493/503 as indicated below. In indicated experiments, adipocytes were labeled with BODIPY FLC_16_ as previously described^33^. Briefly, isolated adipocytes were incubated with BODIPY FLC_16_ at 5 µM in RPMI supplemented with 5 mM glucose and 5% FCS, then washed with pre-warmed PBS, prior to coculture with cancer cells as described above.

### Measure of LDH in the culture medium

The viability of adipocytes cultured in either 2D or 3D was determined by measuring the amount of LDH released into the culture medium using the CyQUANT LDH Cytotoxicity Assay kit (Thermo Fisher Scientific) according to the manufacturer's instructions. The absorbance was measured at 490 nm using a spectrophotometer (MicroQuant, BioTek Instrument Inc). The resulting absorbance was expressed as the percentage of a positive control (set at 100) corresponding to same initial number of adipocytes undergoing 3 cycles of freezing (in liquid nitrogen)/thawing followed by sonication for 5 s.

### Staining of adipocytes and tumor cells

The neutral lipid content of cells was determined by BODIPY® 493/503 staining (Thermo Fisher Scientific). For adipocytes in 2D culture, after recovery of the well content, adipocytes were washed once with PBS and then labeled with BODIPY® 493/503 for 30 min under gentle agitation at room temperature (RT). After staining, adipocytes were washed three times with PBS and then placed in Lab-Tek culture chambers (Dutscher) and analyzed immediately using a confocal microscope. For adipocytes in 3D culture, gels were fixed with 3.7% paraformaldehyde for 1 h at RT, then incubated with 10 µg/mL of BODIPY® 493/503 for 45 min at RT. Single plane (2D) and z-stack fluorescence images were acquired with a confocal laser microscopy system (Confocal TIRF FV1000 Olympus, 10×/NA 0.40 objective, Olympus). Maximum intensity projection was performed using Fiji software (Image J, Bethesda, MD, USA). At least 300 adipocytes from each sample were manually measured with ImageJ. For tumor cells cocultured with adipocytes, cancer cells were labeled with BODIPY® 493/503 (to stain neutral lipids) at 2.5 ng/mL, with rhodamine-phalloidin (to stain actin cytoskeleton) (Abcam) and with DAPI (to stain nucleus) at 1 µM (Thermo Fisher Scientific) as previously described^34^. For tumor cells cocultured with BODIPY FLC16-labeled adipocytes, only rhodamine-phalloidin and DAPI were used. Fluorescence images were acquired with a confocal laser microscopy system (Confocal TIRF FV1000 Olympus, PLAN-APO 60X/NA 1.40 objective, Olympus) and analyzed with Fiji/Image J software.

### Measure of adipocyte lipolytic activity

Lipolytic activity of adipocytes was measured as previously described^32^. Briefly, 100 µL of isolated adipocytes were incubated in 250 µL KRBHA for 3 h at 37 °C with or without 1 µmol/L isoprenaline (Sigma Aldrich) to evaluate stimulated and basal lipolysis. At the end of incubation period, incubation media were collected to quantify the amount of glycerol released using the Free Glycerol Reagent kit (Sigma Aldrich) according to manufacturer’s instructions. For adipocytes embedded in matrix, after medium removal gels were incubated in 250 µL KRBHA with or without isoprenaline and treated as described above.

### Statistics

Statistical analyses were performed by using GraphPad Prism (v8.01). Normal distribution of the data was assessed using the Shapiro Wilk test. For data with normal distribution, Paired t-test or 2-way ANOVA were performed and for data without a normal distribution, Dunn's multiple comparison test was used. P values < 0.05 (*), < 0.01 (**) and < 0.001 (***) were considered significant.

## Results and discussion

### Like SC-Ads, M-Ads rapidly die when cultured in 2D

**Figure 1:**
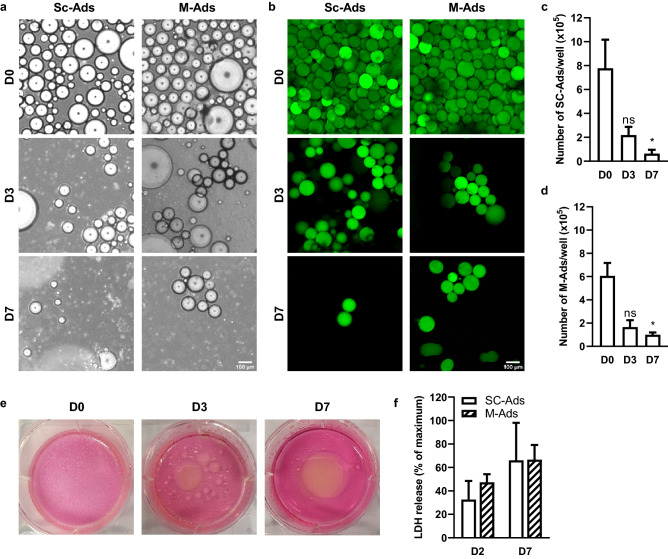
Like SC-Ads, M-Ads rapidly die when cultured in 2D. (**a**) Representative phase-contrast images taken under light microscope of human SC-Ads and M-Ads cultured in 2D for the indicated times; Scale bar, 100 µm. (**b**) Representative images of BODIPY® 493/503 (neutral lipids, green) stained primary SC-Ads and M-Ads in 2D culture for the indicated times; Scale bar, 100 µm. (**c**,**d**) Number of SC-Ads (**c**) and M-Ads (**d**) cultured in 2D over time (n = 3). (**e**) Representative pictures of the culture wells of M-Ads grown in 2D at indicated times points. (**f**) Quantification of LDH release in medium during 2D culture at D2 and D7 for SC-Ads and M-Ads (n = 3). The results are expressed as the percentage of the value obtained when the whole cell population (same cell number than D0) is lysed. The histograms represent mean ± SEM, ns non-significant, *P < 0.05.

As a recently published protocol suggested that primary M-Ads can be cultured in 2D for up to 7 days^23^, we tested this method with M-Ads isolated from lean patients (BMI: 23.4 ± 2.2 kg/m^2^). We used SC-Ads from lean women (BMI: 24.7 ± 3.9 kg/m^2^) as a control since several studies have shown that they cannot be cultured in 2D in a prolonged manner^20–22^. Thus, SC-Ads or M-Ads were put in 6-well plates containing culture medium and kept at 37 °C for 7 days. As shown in Fig. 1a, the number of both M-Ads and SC-Ads decreased sharply in the wells between D0 (when isolated adipocytes were put in culture medium) and D3, where few refringent cells were found under light microscopy. These refringent cells almost disappeared at D7 (Fig. 1a). In order to evaluate their viability, adipocytes were stained using BODIPY® 493/503, a fluorescent dye for neutral lipids (Fig. 1b). As seen at D0, primary adipocytes are spheric cells that contain a unilocular LD considered to be representative of their size. Using this approach, we confirmed the decrease in the number of viable adipocytes for both SC- and M-Ads at D3 and at D7 (Fig. 1b). After counting the unlabeled adipocytes, we found a 70% decrease in the adipocytes number between D0 and D3 for both SC-Ads (Fig. 1c) and M-Ads (Fig. 1d) and a further decrease in cell number was seen between D3 and D7 (Fig. 1c,d). In parallel with this decrease in cell number, oil droplets of increasing size appeared in the wells over time, reflecting lipid release from dying adipocytes into the culture medium, as illustrated by a representative image of cultured M-Ads (Fig. 1e). Finally, to confirm adipocyte death, we quantified LDH in the culture medium, this enzyme being released from cells when plasma membrane integrity is altered. As a positive control, the same number of adipocytes as at the beginning of the culture was subjected to complete lysis by freezing/thawing cycles followed by sonication to evaluate maximal LDH release (value set at 100%). As early as D2, high levels of LDH were detected in the culture medium which further increased at D7, confirming the progressive death of SC- and M-Ads in 2D culture (Fig. 1f). Taken together, our results demonstrated that, like SC-Ads^20–22^, M-Ads were not able to survive in 2D culture and undergo rapid death by necrosis during the first days of culture.

### 3D fibrin matrix preserves adipocytes viability but not lipolytic function at high glucose concentration

**Figure 2:**
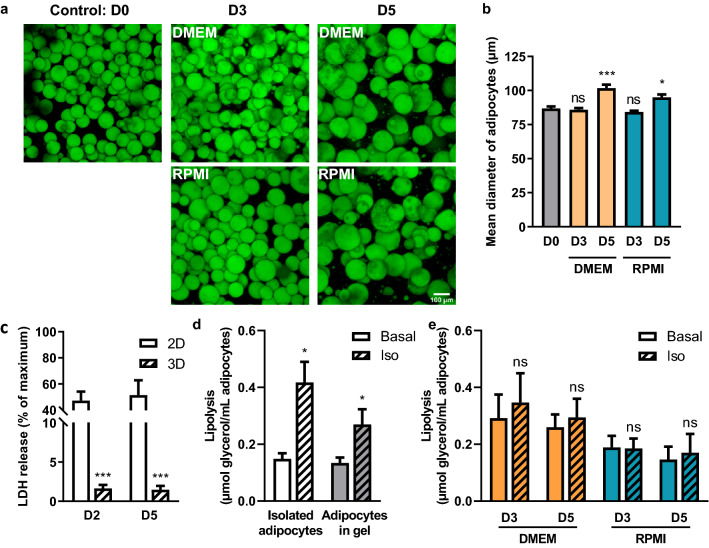
3D fibrin matrix preserves adipocyte viability but not lipolytic function at high glucose concentration. (**a**) Maximum intensity projection of Z-stack acquired through confocal microscopy. Representative images of M-Ads after BODIPY 493/503 staining, cultured in fibrin matrix for the indicated time. Top, cultured with DMEM 25 mM glucose 10% FCS medium, bottom, cultured with RPMI 11 mM glucose, 10% FCS medium; Scale bar, 100 µm. (**b**) Mean diameter of adipocytes according to their culture medium over time. Between 200 and 300 adipocytes were measured per condition (n = 6). (**c**) Quantification of LDH released in medium during culture at the indicated time for M-Ads in 2D (n = 3) or 3D (n = 12) culture. (**d**) Amount of glycerol released after 3 h in the presence or not (basal lipolysis) of isoprenaline (iso) before gel inclusion (isolated adipocytes) or after 3 h in gel (adipocytes in gel) (n = 8). (**e**) Similar experiments were performed with adipocytes cultured in 3D matrix for the indicated time, in RPMI or DMEM (n = 7). Histograms represent mean ± SEM, ns non-significant, *P < 0.05, ***P < 0.001.

One experimental alternative to maintain viable adipocytes for longer times is the use of a 3D matrix. To cultivate M-Ads, we chose fibrinogen to make the gels because of its rapid polymerization in the presence of thrombin^31^. Two different culture media were used, either DMEM containing 25 mM glucose, a medium commonly used to support in vitro adipogenesis^35^, or RPMI 1640 which contains 11 mM glucose. In addition to glucose, other differences in the composition in calcium, phosphate and amino acids between these two media are present that could potentially influence adipocyte viability^36^. We first evaluated the morphology and size of isolated adipocytes at indicated time points (D0, D3 and D5) through BODIPY® 493/503 staining (Fig. 2a,b). Through confocal microscopy, we observed that M-Ads were homogeneously distributed within the matrix, and that their spheric form was maintained for up to 5 days regardless of the medium used (Fig. 2a). During this period, we did not observe any elongated fibroblast-like cells that could result from a process of adipocyte dedifferentiation, attesting that our conditions allowed the maintenance of mature adipocytes in culture (Fig. 2a). At D5, a significant increase in adipocyte size was observed in both media (mean size: 101.7 ± 19.7 µm and 94.9 ± 16.5 µm in DMEM and RPMI respectively) as compared to those freshly embedded in the matrix (D0) (mean size: 86.8 ± 14.4 µm) (Fig. 2b). We concluded that the increase in M-Ads size reflects lipogenesis activity occurring in the presence of glucose, which provides carbon source for FFA synthesis and facilitates FFA esterification for lipid storage^22,37,38^. Absence of adipocyte death was shown by measuring LDH released in the culture medium, which remained very low in 3D compared to 2D culture (Fig. 2c). We then investigated the lipolytic function of adipocytes by using isoproterenol (iso), a β-adrenergic agonist, at doses that ensure maximal lipolytic activation^39,40^. Using freshly isolated cells in suspension as a control, we obtained a significant 2.8-fold increase in glycerol release treated with iso compared to basal condition. This lipolysis activation also occurred in matrix-embedded M-Ads but to a lesser extent (twofold). Nonetheless, at D3 and D5, adipocytes included in matrix were no longer responsive to iso treatment in both DMEM and RPMI (Fig. 2e). A higher basal lipolysis could be noted for cells cultured in DMEM (25 mM glucose) (Fig. 2e) compared to freshly matrix-embedded adipocytes (Fig. 2d) (twofold and 1.7-fold at D3 and D5, respectively), whereas this increase in basal lipolysis was not observed with RPMI that contained less glucose (11 mM) (1.2-fold and onefold at D3 and D5, respectively) (Fig. 2d,e). Such increase in basal lipolysis in the presence of high glucose concentration has been previously reported^41^. The impaired stimulated lipolysis we observed is likely due to the progressive hypertrophy of adipocytes during the culture observed in both media, since stimulated lipolysis has been reported to be negatively correlated to fat cell volume^42,43^. To conclude, we showed that 3D fibrin matrix was able to maintain adipocyte morphology and viability for up to 5 days, however high glucose concentrations in the medium seemed to alter their lipolytic function^36^.

### 3D fibrin matrix preserves adipocyte lipolytic function for up to 5 days at low glucose concentration

**Figure 3:**
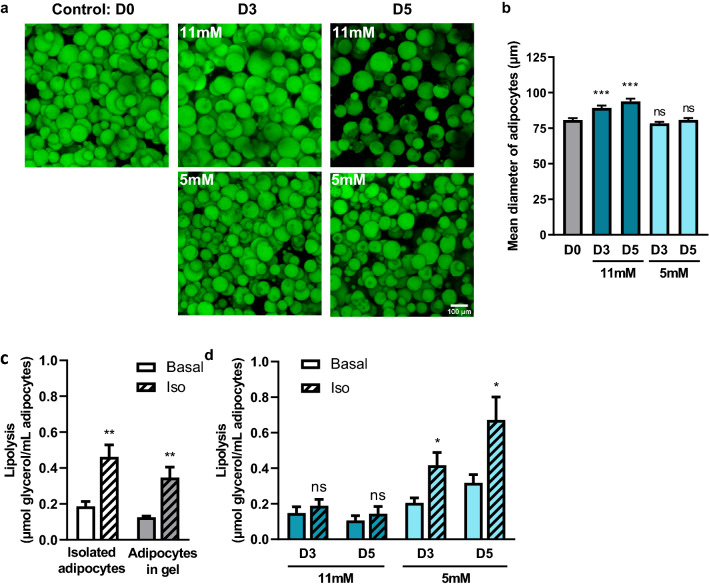
3D fibrin matrix preserves adipocyte lipolytic function for up to 5 days at low glucose concentration. (**a**) Maximum intensity projection of Z-stack acquired through confocal microscopy. Representative images of M-Ads after BODIPY® 493/503 staining, cultured in fibrin matrix for the indicated time. Top, cultured with RPMI 11 mM glucose 10% FCS medium, bottom, cultured with RPMI 5 mM glucose, 10% FCS medium; Scale bar, 100 µm. (**b**) Mean diameter of adipocytes according to their culture medium over time. Between 200 and 300 adipocytes were measured per condition (n = 6). (**c**) Amount of glycerol released after 3 h in the presence or not (basal lipolysis) of isoprenaline (iso) before gel inclusion (isolated adipocytes) or after 3 h in gel (adipocytes in gel) (n = 5). (**d**) Similar experiments were performed with adipocytes cultured in 3D matrix for the indicated time, in RPMI containing 5 mM or 11 mM glucose (n = 5). The histograms represent mean ± SEM, ns non-significant, *P < 0.05, **P < 0.01, ***P < 0.001.

As high glucose concentration seemed to alter M-Ads metabolic function, we lowered the glucose concentration of the RPMI medium to 5 mM to be closer to physiological conditions^36^, and decreased by 1:3 the density of the matrix to improve the release of lipolysis products. As a control, matrix-embedded adipocytes were maintained in parallel in RPMI with 11 mM glucose. As previously observed, the M-Ads were homogeneously distributed within the matrix and maintained their rounded morphology for 5 days regardless of the glucose concentration (Fig. 3a). While M-Ads significantly increased in size when grown in RPMI 11 mM in these set of experiments, no changes in adipocytes size was observed in RPMI 5 mM glucose (Fig. 3b). We then verified the lipolytic function of adipocytes under these culture conditions. With freshly embedded adipocytes in this less dense matrix, the increase in glycerol release after iso stimulation was similar to the one measured with isolated adipocytes (2.8-fold) (Fig. 3c), showing that a less dense matrix improved the release and diffusion of this lipolysis product in the medium. Furthermore, we also evaluated the lipolytic activity of M-Ads at D3 and D5. Significant glycerol release was observed upon iso stimulation at both time points at low glucose condition, contrary to high glucose condition (Fig. 3d), consistent with our hypothesis that the observed progressive hypertrophy (Fig. 3a) could affect lipolytic response. Taken together, our results showed that M-ads embedded in a less dense fibrin matrix and cultured in RPMI with 5 mM glucose retained their size and respond to lipolytic stimulation for up 5 days. These results demonstrated that glucose concentration was a critical parameter for the culture of isolated adipocytes in 3D in order to maintain their metabolic function.

### Our 3D matrix model maintains intact and functional adipocytes in both lean and obese conditions

**Figure 4:**
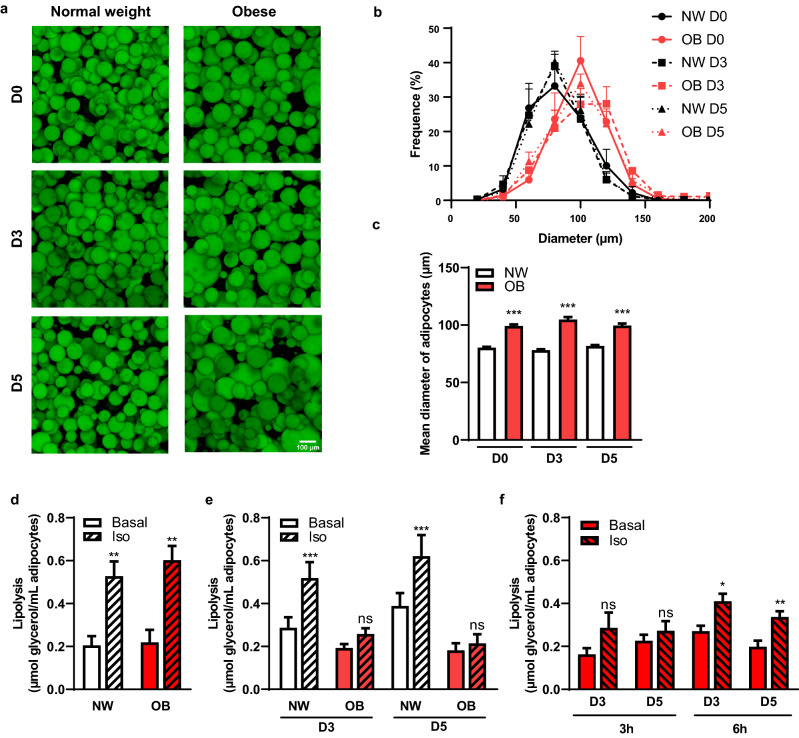
Our 3D matrix model maintains intact and functional adipocytes in both lean and obese conditions. (**a**) Maximum intensity projection of Z-stack acquired through confocal microscopy. Representative images of M-Ads obtained from normal weight or obese patients, after BODIPY® 493/503 staining, cultured in fibrin matrix for the indicated time; Scale bar, 100 µm. (**b**) Distribution of adipocyte diameter (µm) according to their culture condition over time in relative frequency in samples obtained from normal weight (NW) (n = 6) or obese (OB) patients (n = 3). (**c**) Mean diameter of adipocytes (µm) obtained from NW (n = 6) or OB patients (n = 3) at different time point. (**d**) Amount of glycerol released after 3 h in the presence or not (basal lipolysis) of isoprenaline (iso) in adipocytes embedded in gels for NW (n = 9) or OB (n = 6) patients. (**e**) Similar experiments were performed with adipocytes cultured in 3D matrix for the indicated time isolated from NW (n = 16) or OB (n = 10) patients. (**f**) Amount of glycerol released after 3 or 6 h in the presence or not (basal) of isoprenaline (iso) in adipocytes isolated from obese patients (n = 3). Histograms represent mean ± SEM, ns non- significant, *P < 0.05, **P < 0.01, ***P < 0.001.

The main objective of our model was to be able to maintain in culture M-Ads isolated from both lean and obese patients in order to study the impact of obesity on the metabolic symbiosis between adipocytes and tumor cells. A series of samples were obtained from either normal weight (NW) (BMI 21.8 ± 1.9 kg/m^2^, n = 37) or obese (BMI 31.6 ± 2. 8 kg/m^2^, n = 17) patients and used for the different experiments. We first evaluated the morphology and the size of isolated adipocytes at indicated time points (D0, D3 and D5) through BODIPY® 493/503 staining (Fig. 4a,b). Adipocytes isolated from both NW and obese patients showed homogeneous distribution within the matrix and preserved their morphology for up to 5 days (Fig. 4a). Expectedly, size distribution showed that adipocytes from obese patients were significantly larger than those from NW patients (Fig. 4b) and the calculated mean diameter was of 99.2 ± 12.3 µm versus 80.3 ± 8.4 µm respectively at D0 (Fig. 4c). Importantly, these differences in size were maintained during the culture for up to 5 days (Fig. 4c). Both basal and iso-induced lipolysis were similar between NW and obese-isolated adipocytes (Fig. 4d). These results differ from the literature which showed a decreased noradrenaline sensitivity in isolated SC-Ads from obese patients^42,43^. Our data suggest that the lipolytic function of M-Ads induced by β-adrenergic stimulation might not be regulated by obesity in opposition to what is observed in SC-Ads^42,43^. However, we cannot formerly exclude that these results are due to the fact that our patients exhibit only a moderate obesity (BMI: 31.6 ± 2. 8 kg/m^2^) in opposition to studies performed with SC-Ads that included morbidly obese patients (BMI: 43.1 ± 0.7 kg/m^2^)^43^. When obese M-Ads were cultured into matrix, they no longer responded to iso-induced lipolysis after 3 h on D3 and D5, and the basal lipolysis did not increase over time as in NW M-Ads (Fig. 4e). We hypothesized that this absence of response might be due to a defect in the diffusion of iso into the matrix due to the presence of hypertrophic adipocytes and/or to the secretion of excess of ECM molecules by obese adipocytes^44^. Indeed, it has been shown in a 3D culture system that the mechanical constraints induced by a modified ECM obtained from obese AT reduced adipocyte lipolytic function^45^. To circumvent this issue, we exposed obese adipocytes to iso for longer time (6 h instead of 3 h). In these experimental conditions, iso was able to induce glycerol release in obese adipocytes (respectively 1.9- and 1.8-fold at D3 and D5) (Fig. 4f). In conclusion, these results demonstrated that our culture model was able to preserve obese adipocytes integrity and function during 5 days. In addition, our preliminary results showed that adiponectin secretion was decreased by about twofold in samples from obese compared to lean patients and this difference was maintained after 3 days of culture (not shown). These results, although they need to be extended, suggested that this model could also be used to study the endocrine role of adipocytes in their tumor promoting effect.

### The transfer of FFA between adipocytes and BC cells is increased in obesity

**Figure 5:**
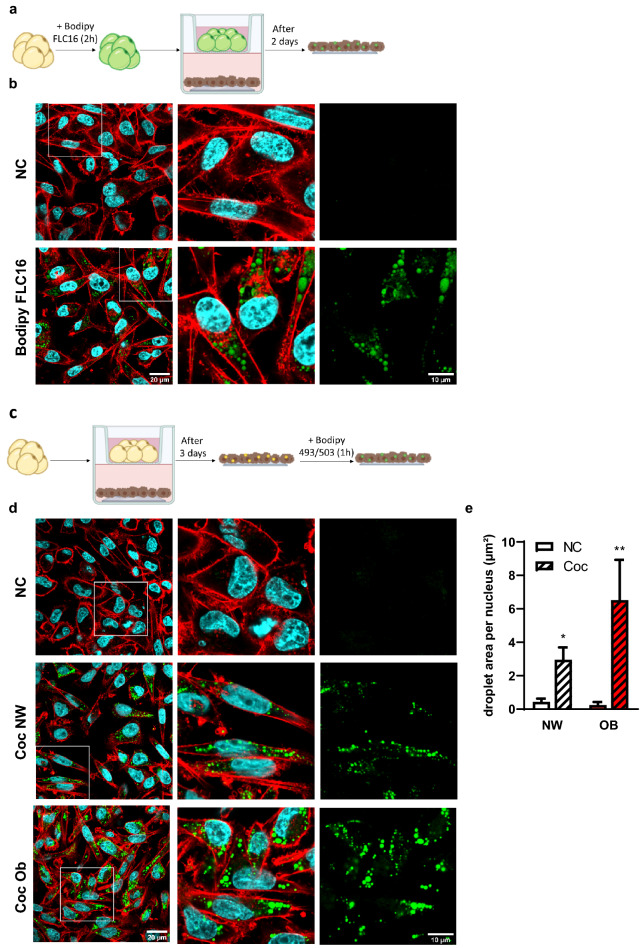
The transfer of FFA between adipocytes and BC cells is increased in obesity. (**a**) Experimental design: Isolated adipocytes (in yellow) were loaded for 2 h with BODIPY FLC_16_ (in green) and cocultured with cancer cells (in brown) for 2 days. (**b**) Representative images of MDA-MB-231 cells cocultured or not (NC) with M-Ads previously loaded with BODIPY FLC_16_ (in green). Actin was labeled with rhodamine-phalloidin (red) and nuclei were labeled with DAPI (blue). The white box in left panels indicates a zoomed crop of this area that is presented in the middle panel. The right panel shows the staining for BODIPY FLC_16_ alone. (**c**) Experimental design used for the 3D coculture followed by BODIPY® 493/503 staining. (**d**) Representative images of MDA-MB-231 cells cocultured or not (NC) for 3 days with adipocytes obtained from normal weight (Coc NW) or obese (Coc Ob) patients after staining with BODIPY® 493/503, (neutral lipids, green), rhodamine-phalloidin (actin, red) and DAPI (nuclei, blue). The white box in left panels indicates a zoomed crop of this area that is presented in the middle panels. The right panel shows the staining for BODIPY® 493/503 alone. (**e**) Quantification of the droplet area per nucleus using ImageJ software in MDA-MB-231 cells cocultured (Coc) or not (NC) for 3 days with adipocytes obtained from NW (n = 10) or obese (OB) patients (n = 4). The histogram represents mean ± SEM *P < 0.05, **P < 0.01.

We then investigated if a transfer of FFA between adipocytes and tumor cells was observed using our 3D culture model. To demonstrate a direct transfer of FFA between M-Ads and breast tumor cells, we used a pulse-chase assay previously developed in our team^33^. We loaded M-Ads obtained from NW patients for 2 h with BODIPY FLC_16_ in suspension, and after being embedded in fibrin matrix, these adipocytes were cocultured for 2 days with cancer cells to monitor the transfer of this fluorescent FFA (Fig. 5a). Large fluorescent LD were detected in cancer cells cocultured with labeled adipocytes as compared to cancer cells grown alone (Fig. 5b). These results demonstrated that cancer cells were able to induce the release of FFA from these mature adipocytes grown in 3D as previously demonstrated using in vitro differentiated models^4,9^. Of note, the fibrin matrix remained macroscopically intact during the coculture. The integrity of adipocytes was also verified at the end of the coculture (data not shown). Fibrin matrices also have the advantage of being less sensitive to numerous proteases secreted by tumor cells compared to Matrigel or collagen-based matrix^46^.

Since we previously demonstrated that hypertrophic adipocytes internalized less lipids than their smaller counterparts^33^, we could not use this pulse-chase assay to compare FFA transfer between M-Ads isolated from NW and obese patients. We therefore used a coculture system between cancer cells and M-Ads isolated from NW and obese patients during 3 days (Fig. 5c). After coculture, tumor cells accumulated numerous LD containing neutral lipids unlike non-cocultured cells and this effect was amplified with obesity (Fig. 5d). The surface area of LD was twice larger when tumor cells were cocultured with adipocytes from obese patients than NW (respectively 6.5 µm^2^/cell and 2.9 µm^2^/cell) (Fig. 5d,e). Increased lipid transfer in obesity did not result in an increase in the number of co-cultured compared to non-cocultured tumor cells (not shown). The effect on other aspects of tumor progression such as survival, migratory and invasive properties are under investigation. Taken together, our results showed that M-Ads cultured in a fibrin matrix released FFA that were taken up by tumor cells and that this effect was amplified with obesity.

## Conclusion

Obesity has been shown to negatively affect BC prognosis^11,12^. Yet, biological mechanisms underlying this effect are still largely unknown. An emerging hypothesis is that the transferred FFA between adipocytes and tumor cells may be quantitatively and qualitatively altered under obese conditions, therefore contributing to tumor progression^4^. To answer these questions, establishing new culture methods adapted to M-Ads obtained from both lean and obese patients is fundamental, as changes in lipid content (qualitative and quantitative) under obesity across different adipose tissues has been highlighted by recent studies^19,30^. Here, we showed that, like other isolated adipocytes such as SC-Ads, M-Ads rapidly die when grown in 2D, in contradiction with a recently published protocol^23^. To circumvent this issue, we set up a 3D culture model using fibrin matrix which allows homogeneous distribution of the embedded adipocytes and the preservation of their integrity for up to 5 days in both lean and obese conditions. One of the key findings of our study is that culturing M-Ads in physiological glucose concentration is mandatory to prevent lipogenesis during the time of culture, and preserve their lipolytic function. To date, most of the proposed systems have been using media containing at least 11 mM glucose^24,26,27^. By adding matrix embedded adipocytes into transwell inserts, we demonstrate the ability to coculture them with cancer cells (without altering the matrix) and highlight the presence of a metabolic crosstalk between these cells. In addition, our preliminary results indicate that this crosstalk is amplified by obesity. This coculture system will further allow us to study the nature of lipids liberated by M-Ads upon tumor secretions, and their impact on tumor aggressiveness in both lean and obese conditions. Given the important questions remaining about the role of the immune system in the obesity-driven BC progression^14^, this model could also be useful to study the metabolic crosstalk between adipocytes and immune cells including macrophages. As a conclusion, our model is a valuable translational new tool to study mechanisms underlying obesity–BC relationship that remains unclear.

## Data Availability

The data that support the findings of this study are available from the corresponding author, [CM], upon request.
